# Seasonal and environmental drivers of antibiotic resistance and virulence in *Escherichia coli* from aquaculture and their public health implications

**DOI:** 10.1038/s41598-025-98498-8

**Published:** 2025-05-08

**Authors:** Aya El Badawy, Dalia Hamza, Zeinab Ahmed, Maha A. Sabry

**Affiliations:** Department of Zoonoses, Faculty of Veterinary Medicine, Cairo University, PO Box 12211, Giza, Egypt

**Keywords:** Seasonal variations, *E. coli*, Virulence genes, Antibiotic resistance genes, Fish farms, Egypt, Antimicrobials, Environmental microbiology

## Abstract

Aquaculture is increasingly impacted by environmental stressors such as temperature and pH fluctuations, which influence the proliferation and antibiotic resistance of *Escherichia coli* (*E. coli*). This study investigates the effects of these factors on the prevalence, virulence, and antibiotic resistance of *E. coli* isolated from aquaculture environments in Egypt, with a focus on public health implications. A total of 328 *Oreochromis niloticus* (Nile tilapia) samples were collected from Egyptian fish farms over five sampling periods, representing different seasonal conditions. *E. coli* was isolated and identified using selective culture methods and biochemical tests. Molecular characterization was conducted via polymerase chain reaction (PCR) to detect diarrheagenic *E. coli* pathotypes (*st, lt, eaeA, bfpA, stx1, stx2*). Additionally, PCR was utilized to screen for β-lactamase and carbapenemase resistance genes. Water parameters, including temperature and pH, were recorded, and their correlation with bacterial prevalence, virulence, and antibiotic resistance profiles were analyzed. A high prevalence of *E. coli* (92.68%) was observed, with a significant correlation between bacterial occurrence and elevated water temperatures. Diarrheagenic *E. coli* was detected in 82.1% of samples, with enterotoxigenic *E. coli* (ETEC) being the most common pathotype. Some isolates harbored multiple virulence genes, indicating hybrid strains. Resistance genes such as *bla*_TEM_, *bla*_CTX-M_, and *bla*_OXA-48_ were widely distributed, particularly during warmer months and at neutral pH levels. Groups with elevated water temperatures exhibited a higher prevalence of antibiotic-resistant isolates, often harboring multiple resistance genes. This study highlights the significant role of environmental stressors in influencing the prevalence, pathogenicity, and antibiotic resistance profiles of *E. coli* in aquaculture systems. The findings emphasize the need for continuous monitoring and improved biosecurity measures to mitigate the risks associated with MDR *E. coli* in aquaculture, ensuring food safety and protecting public health.

## Introduction

Climate change has become a critical and widely discussed global issue^1^, with significant implications for various sectors, including aquaculture^2^. It impacts aquaculture by altering water quality, threatening species viability, and risking sustainable production^3^. Higher global temperatures are directly linked to increased water temperatures, which elevate mortality rates in aquatic species^4^. Water quality fluctuations, such as CO₂-induced acidification, further compromise fish health by disrupting pH balance^5^. Combined with oxygen deprivation, these stressors reduce fish growth, reproduction, and survival^6,7^. Climate change also exacerbates existing aquaculture stressors and contributes to the emergence of zoonotic diseases^8^. The World Health Organization has recognized that these environmental changes contribute to the spread of infectious diseases, posing risks not only to aquatic species but also to human health through water-related diseases, which are projected to rise due to climate change^9,10^.

*Escherichia coli* poses a significant threat to aquaculture, leading to economic losses and public health risks. Pathogenic *E. coli* strains cause symptoms such as sores, hemorrhages, and slowed growth rates in fish^11^, with infections influenced by environmental changes like rising water temperatures and altered precipitation patterns, which favor bacterial proliferation in aquatic systems^1,12^. In aquaculture, *E. coli* enters through contaminated water or feed, forms biofilms, invades host tissues, and releases toxins, leading to intestinal damage and systemic infections^11^. The implications of *E. coli* infections extend to public health, as contamination can cause foodborne illnesses ranging from mild gastrointestinal symptoms to severe conditions like hemolytic uremic syndrome (HUS), particularly affecting vulnerable populations such as young children and the elderly^13^. Pathogenic *E. coli* strains are classified into different pathotypes based on their virulence factors, infection mechanisms, and clinical effects. Key diarrheagenic *E. coli* (DEC) pathotypes include enteropathogenic *E. coli* (EPEC), enterotoxigenic *E. coli* (ETEC), and Shiga toxin-producing *E. coli* (STEC), each possessing specific genes that facilitate adhesion, toxin production, and disease development^14^. Virulence factors such as adhesins (fimbriae, intimin), toxins (Shiga-like toxins, hemolysins), invasins, and siderophores enable *E. coli* to colonize fish, disrupt cellular functions, and cause infections^15–17^.

Enterotoxigenic *Escherichia coli* (ETEC) produces heat-stable (ST) and heat-labile (LT) enterotoxins, leading to watery diarrhea, traveler’s diarrhea, and infantile diarrhea in developing countries. Shiga toxin-producing *E. coli* (STEC), also known as enterohemorrhagic *E. coli* (EHEC), is characterized by the *stx* and *eaeA* genes, which encode Shiga toxins (Stx1 and Stx2) associated with severe outcomes like HUS^15–17^. Enteropathogenic *E. coli* (EPEC) expresses the *eaeA* gene, crucial for attachment and effacement lesions, and the *bfpA* gene, encoding the bundle-forming pilus (BFP) essential for intestinal adherence^15–17^. EPEC is further classified into typical (tEPEC) and atypical (aEPEC) based on the presence of the adherence factor plasmid (pEAF)^18^. Temperature plays a pivotal role in regulating the transcription of virulence genes in *E. coli*, influencing its pathogenicity under varying environmental conditions^19^.

Antimicrobial-resistant (AMR) in aquaculture is a significant public health concern, primarily driven by the excessive use of antibiotics in fish farming, which fosters the emergence of multidrug-resistant (MDR) strains^20^. Intensive aquaculture systems act as reservoirs for these bacteria, threatening fish health and enabling their transmission to humans through the food chain, leading to hard-to-treat illnesses^20–22^. Rising temperatures due to climate change significantly contribute to the proliferation of antibiotic resistance in ecosystems by increasing selective pressure on bacteria and facilitating horizontal gene transfer of resistance genes^10,23^. Extended-spectrum β-lactamases (ESBLs) and carbapenemases represent significant challenges in managing antibiotic resistance in *E. coli*^24^. ESBLs, such as *bla*_TEM_*, bla*_SHV_*, bla*_CTX-M*,*_ and *bla*_OXA-1_, hydrolyze a broad range of β-lactam antibiotics^25^, while carbapenemases, including *bla*_KPC_, *bla*_NDM_, *bla*_OXA-48_ and *bla*_VIM,_ confer resistance to carbapenems, which are often used as a last-line treatment for MDR Gram-negative infections^26^.

Recent studies have often neglected the influence of environmental factors on aquaculture habitats and the organisms residing within them. Specifically, there is a lack of comprehensive knowledge regarding how environmental variables such as temperature and pH affect the distribution of antibiotic-resistant genes in *E. coli* isolates from Egyptian fish farms. This study addresses this gap by investigating the combined impact of environmental stressors such as temperature and pH on the prevalence of *E. coli* pathotypes, their virulence factors, and antibiotic-resistance genes in fish farms in Egypt. Additionally, the study evaluates the potential zoonotic risks posed by consuming fish contaminated with resistant pathogenic *E. coli*.

## Materials and methods

### Sample collection and processing

A total of 328 Nile Tilapia (*Oreochromis niloticus*) were randomly collected from two fish farms with similar environmental conditions across five sampling periods: winter, spring, early summer, summer, and autumn. Environmental parameters, including water temperature and pH levels, were routinely monitored onsite in the fishponds using specialized portable devices. Water temperature was measured using aquarium thermometers with LCD panels for precise readings, while pH levels were determined using a Fisher Scientific pH meter (Waltham, Massachusetts, USA), following the manufacturer’s guidelines. Measurements were taken from multiple locations within each pond to ensure representative and reliable data, which were subsequently analyzed for seasonal trends.

The samples were grouped into five categories based on seasonal environmental conditions: Group 1 (winter: 22 °C, pH 7.5), Group 2 (spring: 28 °C, pH 8), Group 3 (early summer: 35 °C, pH 6.5), Group 4 (summer: 39 °C, pH 5.5), and Group 5 (autumn: 37 °C, pH 6). These samples were further organized into 82 pools, each consisting of four fish. The seasonal distribution of the pools included 15 pools in winter, 18 in spring, 18 in early summer, 16 in summer, and 15 in autumn. The samples were transported under sterile and refrigerated conditions to prevent contamination and maintain their suitability for laboratory analysis.

For each pool, the skin surface, intestinal tract, and muscle tissues of the fish were examined. Skin samples were collected by swabbing various areas of the fish’s surface with sterile cotton swabs. To ensure sterility, a red-hot scalpel was used to disinfect the exterior of the fish before excising portions of the gut and muscle tissue for analysis.

In this study, tilapia fish were euthanized using rapid cooling in water below 4 °C to minimize distress. Following euthanasia, the fish were disposed of by incineration.

### Isolation and identification of *E. coli*

The collected pooled samples were placed into a sterile tube containing fresh tryptic soy broth (HiMedia, India) and incubated overnight at 37 °C, after enrichment a loopful of the broth was streaked onto MacConkey agar (Oxoid, Hampshire, UK), and Eosin methylene blue agar (EMB) (Oxoid, Hampshire, UK) and incubated aerobically at 37 °C for 24–48 h. Colonies exhibiting characteristic pink coloration on MacConkey agar and metallic green shiny on EMB were selected and sub-cultured to obtain pure isolates. *E. coli* identification was based on colonial morphology and Gram staining, followed by further characterization using conventional biochemical tests, including the IMViC test (Indole, Methyl Red, Voges-Proskauer, and Citrate), according to methods described by Tille^27^.

### Extraction of the genomic DNA

Genomic DNA was extracted from the *E. coli* isolates using a conventional boiling process according to Lesiani et al.^28^. The DNA concentration and purity of each sample were determined using a NanoDrop Spectrophotometer. The extracted DNA was preserved at -20 °C until ready for use.

### Molecular identification and characterization of virulence genes in *E. coli* isolates

All *E. coli* isolates were analyzed using multiplex PCR to identify six virulence genes (*eaeA, bfpA, stx1, stx2, st, lt*) according to Prakasan et al.^29^ and Samir et al.^30^. Bacterial DNA was amplified in a 25 µL PCR reaction with 12.5 µL of Emerald Amp MAX PCR master mix (Takara, Japan), 3 µL of template DNA from each isolate, 0.5 µL of each primer (10 pmol/µL; Metabion, Germany), and PCR-grade water to reach the final volume. The sequences of the primers, amplicon sizes, and PCR conditions are demonstrated in Table 1.

**Table 1:** The sequence of oligonucleotide primers and their PCR conditions used in the current study.

Target gene (bp)	Primer sequence (5ʹ–3ʹ)	Cycling condition	References
Virulence genes of *E. coli*			
***eaeA*** (891 bp)	**F**: GTGGCGAATACTGGCGAGACT **R**: CCCCATTCTTTTTCACCGTCG	94℃ for 1 min;30 cycles (94℃ for 30 s, 55℃ for 30 s, and 72℃ for 1 min), 72℃ for 10 min	^29^
***bfpA*** (326 bp)	**F**: AATGGTGCTTGCGCTTGCTGC **R**: GCCGCTTTATCCAACCTGGTA		
***stx1*** (370 bp)	**F**:AAATCGCCATTCGTTGACTACTTCT **R**:CAGTCGTCACTCACTGGTTTCATCA		
***stx2*** (283 bp)	**F**:TGCCATTCTGGCAACTCGCGATGCA **R**: GGATCTTCTCCCCACTCTGACACC		
***st*** (190 bp)	**F**: ATTTTTCTTTCTGTATTGTCTT **R**: CACCCGGTACAAGCAGGATT		^30^
***lt*** (450 bp)	**F**: GGC GAC AGA TTA TAC CGT GC **R**: CGG TCT CTA TAT TCC CTG TT		
β lactamase (ESBLs)-encoding genes			
***bla***_**TEM**_ (445 bp)	**F:**CGCCGCATACACTATTCTCAGAATGA **R:**ACGCTCACCGGCTCCAGATTTAT	95 ºC for 5 min; 30 cycles (94 ºC for 30 s, 62 ºC for 90 s, 72 ºC for 60 s), 72 ºC for 10 min	^32,33^
***bla***_**SHV**_ (237 bp)	**F:**CTTTATCGGCCCTCACTCAA **R:**AGGTGCTCATCATGGGAAAG		
***bla***_**CTX-M**_ (593 bp)	**F:**ATGTGCAGYACCAGTAARGTKATGGC **R:**TGGGTRAARTARGTSACCAGAAYC AGC GG		
***bla***_**OXA-1**_ (813 bp)	**F:**ACA CAA TAC ATA TCA ACT TCG C **R:**AGT GTG TTT AGA ATG GTG ATC		
Carbapenemase-encoding genes			
***bla***_**KPC**_ (882 bp)	**F:**ATG TCA CTG TAT CGC CGT CT **R:** TTT TCA GAG CCT TAC TGC CC	95 ºC for 5 min; 30 cycles (94 ºC for 1 min, 55 ºC for 1 min , 72 ºC for 2 min), 72 ºC for 10 min	^36^
***bla***_**NDM**_ (621 bp)	**F:**GGT TTG GCG ATC TGG TTT TC **R:**CGG AAT GGC TCA TCA CGA TC		
***bla***_**VIM**_ (261 bp)	**F:**AGT GGT GAG TAT CCG ACAG **R:**ATG AAA GTG CGT GGA GAC	95 ºC for 5 min; 35 cycles (94 ºC for 30 Sec, 55 ºC for 30 Sec, 72 ºC for 1 min), 72 ºC for 10 min	^35^
***bla***_**OXA-48**_ (283 bp)	**F :**GCTTGATCGCCCTCGATT **R:** GATTTGCTCCGTGGCCGAAA	94 ºC for 10 min; 30 cycles (94 ºC for 40 Sec, 60 ºC for 40 Sec, 72 ºC for 1 min), 72 ºC for 7 min	^34^

Diarrhoeagenic *E. coli* strains were categorized into different pathotypes based on the presence of these virulence genes. The ETEC pathotype expresses the genes *st* and *lt*. The EPEC pathotype expresses *eaeA* and *bfpA* genes, whereas STEC expresses *stx1*, *stx2* genes. Strains that tested positive for genes expressed by various pathotypes were categorized as hybrids^31^.

### Molecular detection of β-lactamase and carbapenem resistance-encoding genes

Multiplex-PCR was performed to detect β-lactamase-encoding genes *bla*_TEM_*, bla*_SHV_*, bla*_CTX-M*,*_ and *bla*_OXA-1_ employing specific primers mentioned in Table 1. The reaction was carried out following Monstein et al.^32^ and Djeffal et al.^33^.

Additionally, both multiplex and uniplex PCR assays were employed to identify carbapenemase-encoding genes in *E. coli* isolates. Uniplex PCR was used to screen for the presence of the *bla*_OXA-48_ and *bla*_VIM_ genes, following the protocols outlined by Dallenne et al.^34^ and Li et al.^35^. Meanwhile, multiplex PCRwas performed to identify the *bla*_KPC_ and *bla*_NDM_ genes according to Mohammed et al.^36^. The PCR mixtures were run in a total volume of 25 µl, with 5 µl of template DNAfrom each isolate, 12.5 µl of Emerald Amp MAX PCR master mix (Takara, Japan), 0.5 µl of each primer (10 pmol/µl; Metabion, Germany), and PCR-grade water to get the volume up to 25 µl. The specific oligonucleotide of each primer and PCR amplification conditions are detailed in Table 1.

### Statistical analysis

Pearson’s correlation coefficient (*r*) and regression analysis (*R*^2^) were used to assess the relationship between the variables under study. All statistical tests were performed using PASW Statistics, Version 18.0 (SPSS Inc., Chicago, IL, USA). Statistical significance was defined as a *P* value less than 0.05.

## Results

### Occurrence of *E. coli* in different environmental fish groups

A total of 76 confirmed *E. coli* isolates were obtained from 82 pooled fresh fish samples collected from two fish farms in Egypt, resulting in an overall prevalence of 92.68%. The occurrence of *E. coli* varied somewhat throughout the seasons. The seasonal variation in the frequency of *E. coli* is summarized in Table 2.

**Table 2:** Seasonal distribution and occurrence of *E. coli* in fish across different environmental groups.

Different environmental groups	Sampling period	No. of Examined fish	Positive for *E. coli*	
			No	%
Group 1	Winter	15	11	73.33
Group 2	Spring	18	17	94.44
Group 3	Early Summer	18	17	94.44
Group 4	Summer	16	16	100
Group 5	Autumn	15	15	100
Total	-	82	76	92.68

**Fig. 1:**
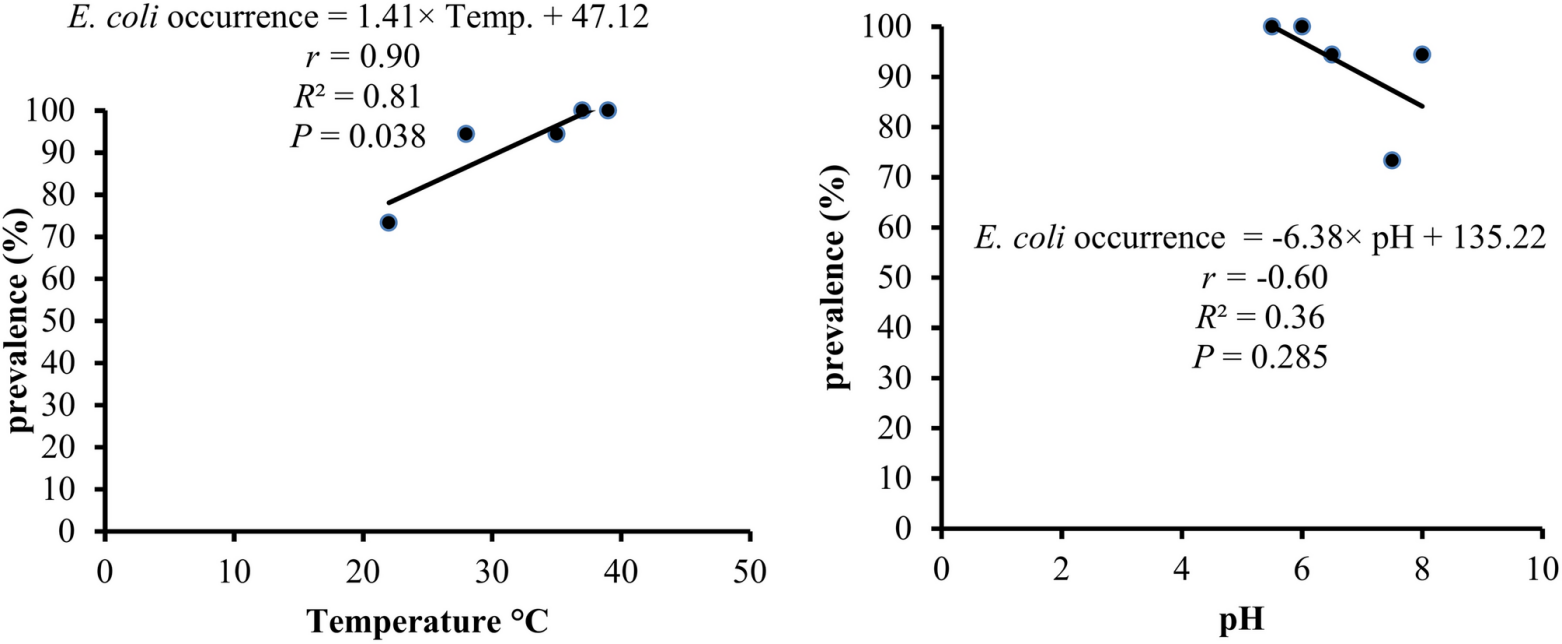
Relation between occurrence of *E. coli* and fishpond temperature and water pH.

The relationship between the occurrence of *E. coli* and fishpond temperature and water pH is illustrated in Fig. 1. A strong positive correlation was observed between the occurrence of *E. coli* and fishpond temperature (*r* = 0.90, *P* = 0.038). For every 1 °C increase in water temperature, the occurrence of *E. coli* increased by 1.41 (*P* = 0.038, *R*^2^ = 0.81). Conversely, a moderate negative association was noted between the occurrence of *E. coli* and water pH (*r* = -0.60, *P* = 0.285), with a decrease in pH associated with a 6.38 increase in the occurrence of *E. coli* (*P* = 0.285, *R*^2^ = 0.36).

### Occurrence of diarrhoeagenic *E. coli* pathotypes and their associated virulence genes

A total of 69 DEC isolates were obtained from 82 pooled fresh fish samples collected from two fish farms in Egypt, resulting in an overall prevalence of 84.1%. The occurrence of DEC pathotypes and their associated virulence genes is summarized in Table 3.

**Table 3:** Distribution of diarrheagenic *E. coli* Pathotypes and associated virulence genes across different environmental groups.

Groups (sampling period)	Diarrhoeagenic *E. coli* (+)	ETEC		STEC		tEPEC	aEPEC		ETEC + STEC	ETEC + aEPEC	ETEC + STEC + aEPEC
		*st*	*st* + *lt*	*stx 1*	*stx 2*	*eaeA* + *bfpA*	*eaeA*	*bfpA*			
Group 1 (Winter)	9	8/11		1/11		0/11	2/11		0	2	0
		6	2	0	1	0	0	2			
Group 2 (Spring)	12	11/17		3/17		0/17	1/17		3	0	0
		8	3	0	3	0	1	0			
Group 3 (Early Summer)	17	17/17		7/17		0/17	3/17		7	3	0
		7	10	2	5	0	2	1			
Group 4 (Summer)	16	15/16		8/16		1/16	5/16		8	5	0
		14	1	1	7	1	2	3			
Group 5 (Autumn)	15	15/15		11/15		0/15	3/15		9	1	2
		13	2	3	8	0	1	2			

Enterotoxigenic *E. coli* (ETEC) was widely distributed across all seasons, with particularly high prevalence in warmer conditions. PCR analysis of virulence genes revealed that the most frequently detected gene was *st*, with no strains carrying only the *lt* gene; instead, it was found in combination with *st* across all groups.

Additionally, other pathotypes such as STEC, aEPEC, and tEPEC were identified. STEC showed low prevalence in winter but increased during the summer and autumn, with the *stx2* gene being more prevalent than *stx1*. Similarly, aEPEC was predominantly found in summer while tEPEC was rare, with only one isolate containing the tEPEC gene identified in summer.

Hybrid pathotypes were also observed, with some *E. coli* isolates exhibiting characteristics of two distinct pathotypes. Notably, Group 5, representing autumn with environmental conditions of 37 °C and pH 6, exhibited a combination of three *E. coli* pathotypes: ETEC, STEC, and aEPEC.

### Distribution of β-lactamase and carbapenemase-encoding genes

**Fig. 2:**
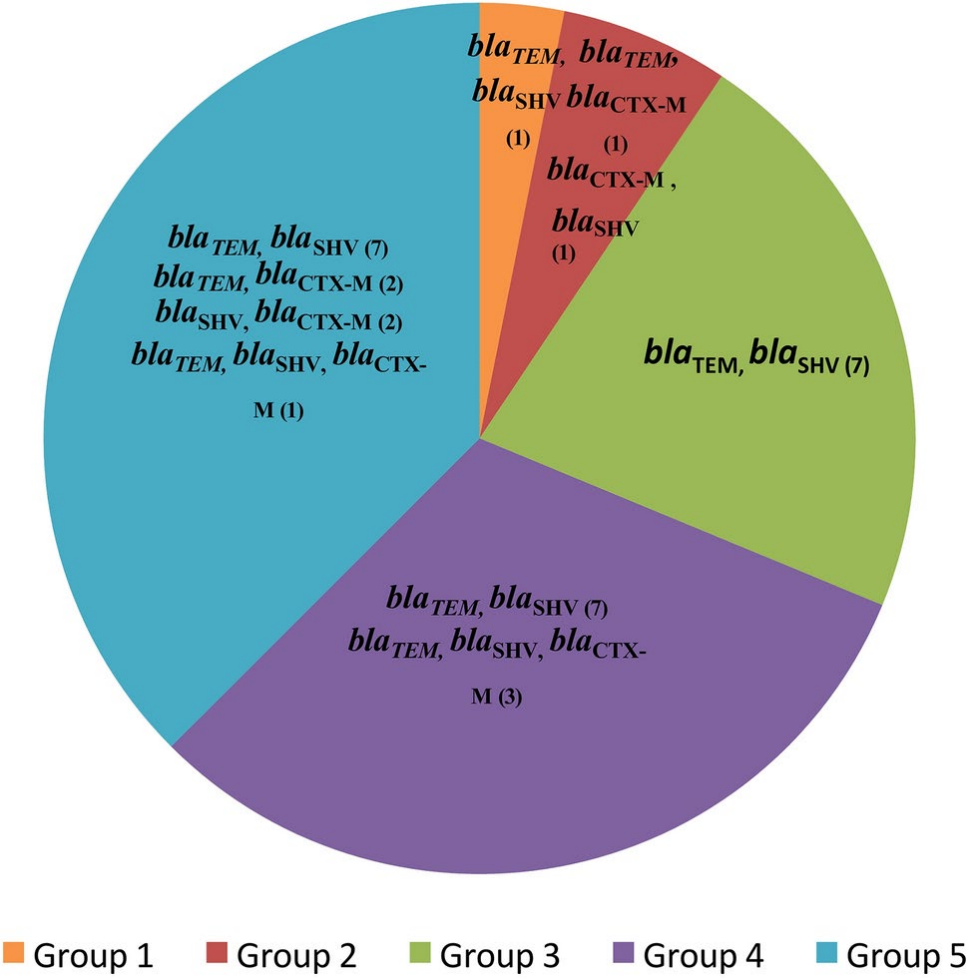
The occurrence of mixed β lactamase (ESBLs)—encoding genes among *E. coli* isolates from fish farms in Egypt across different environmental groups.

**Fig. 3:**
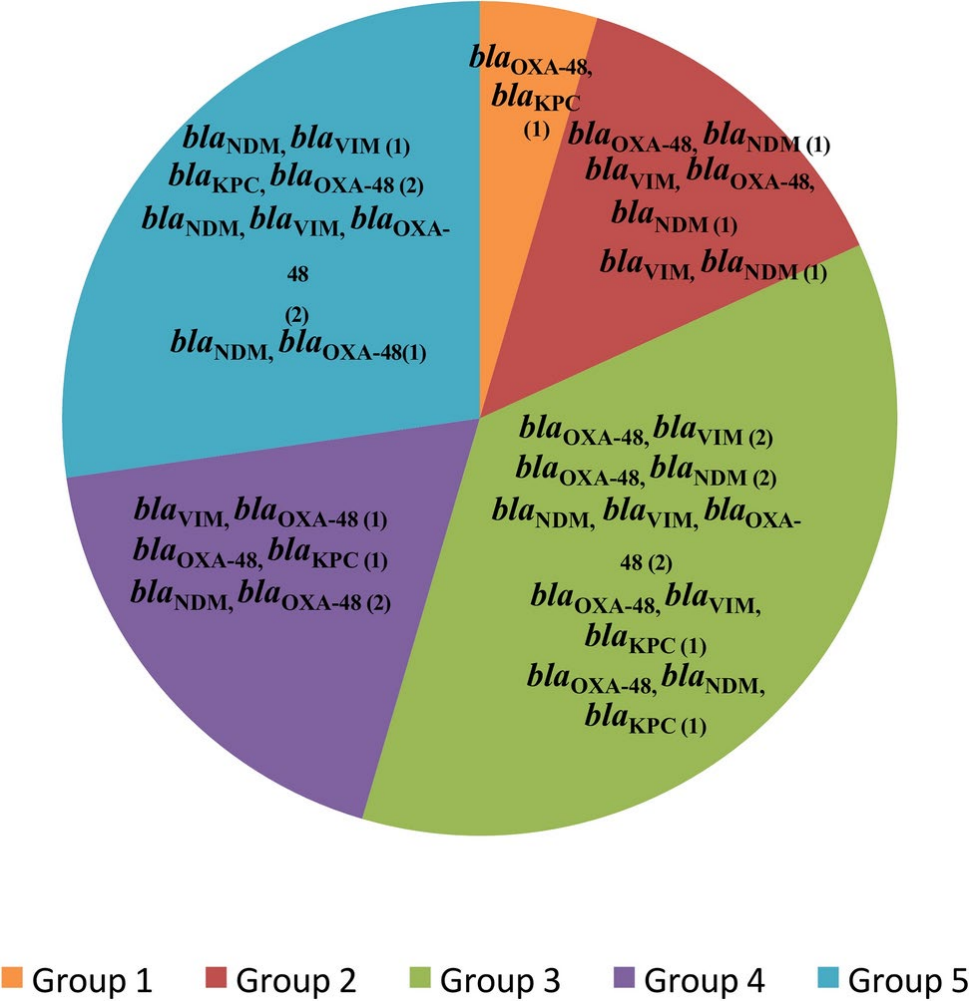
The occurrence of mixed Carbapenemase-encoding genes among *E. coli* isolates from fish farms in Egypt across different environmental groups.

The distribution of carbapenemase-encoding and β-lactamase-encoding genes (ESBLs) in *E. coli* isolates is summarized in Table 4, Figs. 2, and 3. The results indicate that the summer season (39 °C, pH 5.5) and autumn season (37 °C, pH 6) exhibited the highest prevalence of *E. coli* isolates carrying β-lactamase genes. Additionally, early summer (35 °C, pH 6.5), summer (39 °C, pH 5.5), and autumn (37 °C, pH 6) showed the highest proportions of *E. coli* isolates harboring carbapenemase genes.

**Table 4:** The occurrence of β lactamase (ESBLs) and carbapenemase—encoding genes of all *E. coli* isolates from fish farms in Egypt across different environmental groups.

Groups (sampling period)	Isolates carry β-lactamase-encoding genes (ESBLs)				Isolates carry carbapenemase-encoding genes			
	*bla*_TEM_	*bla*_SHV_	*bla*_CTX-M_	*bla*_OXA-1_	*bla*_KPC_	*bla*_NDM_	*bla*_VIM_	*bla*_OXA-48_
Group 1 (Winter)	8/11				3/11			
	5 (45.45%)	2 (18.18%)	2 (18.18%)	0 (0%)	1 (9.09%)	0 (0%)	1 (9.09%)	2 (18.18%)
Group 2 (Spring)	12/17				6/17			
	8 (47.06%)	3 (17.65%)	3 (17.65%)	0 (0%)	2 (11.76%)	3 (17.65%)	3 (17.65%)	2 (11.76%)
Group 3 (Early Summer)	12/17				13/17			
	9 (52.94%)	7 (41.18%)	3 (17.65%)	0 (0%)	2 (11.76%)	7 (41.18%)	7 (41.18%)	9 (52.94%)
Group 4 (Summer)	16/16				7/16			
	16 (100%)	10 (62.5%)	3 (18.75%)	0 (0%)	1 (6.25%)	3 (18.75%)	2 (12.5%)	5 (31.25%)
Group 5 (Autumn)	15/15				12/15			
	13 (86.67%)	10 (66.67%)	5 (33.33%)	0 (0%)	3 (20%)	6 (40%)	6 (40%)	5 (33.33%)

Among the β-lactamase (ESBL)-encoding genes, *bla*_TEM_ was significantly more prevalent during the summer season, while *bla*_SHV_ and *bla*_CTX-M_ showed variable distributions, with their highest abundance recorded in Group 5 (autumn). Notably, *bla*_OXA-1_ was absent across all seasons. Groups 4 (summer) and 5 (autumn), characterized by elevated water temperatures, also had the largest proportion of isolates carrying multiple β-lactamase-encoding genes, as illustrated in Fig. 2.

For carbapenemase-encoding genes, *bla*_OXA-48_, *bla*_VIM_, and *bla*_NDM_ were most frequently detected during early summer (Group 3), while *bla*_KPC_ was less common but observed across all seasons, with the highest prevalence in autumn (Group 5). Group 3 (early summer) exhibited the highest occurrence of isolates carrying multiple carbapenemase-encoding genes, as depicted in Fig. 3.

The distribution of isolates co-harboring both β-lactamase- and carbapenemase-encoding genes across the different environmental fish groups is shown in Table 5. As water temperatures increased, Groups 3 (early summer), 4 (summer), and 5 (autumn) displayed a variety of gene combinations.

**Table 5:** The occurrence of mixed β lactamase and carbapenemase- encoding genes among *E.coli* isolates from fish farms in Egypt across different environmental groups.

Different environmental fish groups	No. of isolates	Mixed β lactamase and carbapenemase- encoding genes
Group 1 (Winter)	1	*bla*_CTX-M,_ *bla*_VIM_
	1	*bla*_*TEM,*_* bla*_OXA-48_
	1	*bla*_*TEM,*_* bla*_SHV,_ *bla*_OXA-48,_ *bla*_KPC_
Group 2 (Spring)	1	*bla*_*TEM,*_* bla*_CTX-M_
	1	*bla*_*TEM,*_* bla*_KPC_
	1	*bla*_*TEM,*_* bla*_OXA-48,_ *bla*_NDM_
	1	*bla*_VIM*,*_* bla*_OXA-48,_ *bla*_NDM_
	1	*bla*_CTX-M,_ *bla*_SHV_,* bla*_VIM*,*_* bla*_NDM_
Group 3 (Early Summer)	1	*bla*_OXA-48,_ *bla*_VIM_
	1	*bla*_CTX-M,_ *bla*_NDM_
	1	*bla*_CTX-M_* bla*_VIM_
	1	*bla*_*TEM*,_ *bla*_OXA-48,_ *bla*_NDM_
	1	*bla*_*TEM*,_ *bla*_SHV,_ *bla*_NDM_
	1	*bla*_*TEM,,*_* bla*_SHV_,* bla*_VIM_
	1	*bla*_*TEM,*_* bla*_SHV_,* bla*_VIM,_ *bla*_OXA-48_
	1	*bla*_*TEM,*_* bla*_NDM_, *bla*_VIM,_ *bla*_OXA-48_
	1	*bla*_*TEM,*_* bla*_SHV_, *bla*_OXA-48,_ *bla*_NDM_
Group 4 (Summer)	2	*bla*_*TEM,*_* bla*_*SHV*_
	1	*bla*_*TEM,*_* bla*_*SHV,*_* bla*_*VIM*_
	1	*bla*_*TEM,*_* bla*_*SHV,*_* bla*_*CTX-M*_
	1	*bla*_*TEM,*_* bla*_*SHV,*_* bla*_*VIM,*_* bla*_*OXA-48*_
	1	*bla*_*TEM,*_* bla*_*SHV,*_* bla*_*OXA-48*_
	1	*bla*_*TEM,*_* bla*_*SHV,*_* bla*_*NDM*_
	1	*bla*_*TEM,*_* bla*_*SHV,*_* bla*_*OXA-48,*_* bla*_*KPC*_
	2	*bla*_*TEM,*_* bla*_*SHV,*_* bla*_*CTX-M,*_* bla*_*NDM,*_* bla*_*OXA-48*_
Group 5 (Autumn)	1	*bla*_*TEM,*_* bla*_*VIM*_
	4	*bla*_*TEM,*_* bla*_*SHV*_
	1	*bla*_*TEM,*_* bla*_*NDM,*_* bla*_*CTX-M*_
	1	*bla*_*TEM,*_* bla*_*VIM,*_* bla*_*CTX-M*_
	2	*bla*_*SHV,*_* bla*_*VIM,*_* bla*_*CTX-M*_
	1	*bla*_*TEM,*_* bla*_*SHV,*_* bla*_*NDM*_
	1	*bla*_*TEM,*_* bla*_*SHV,*_* bla*_*NDM,*_* bla*_*VIM*_
	2	*bla*_*TEM,*_* bla*_*SHV,*_* bla*_*VIM,*_* bla*_*KPC*_
	1	*bla*_*TEM,*_* bla*_*SHV,*_* bla*_*CTX-M,*_* bla*_*NDM,*_* bla*_*VIM*_

### The occurrence of pathotypes and antibiotic resistance genes among *E. coli* isolates

ETEC was the most frequently identified *E. coli* pathotype across all seasons, demonstrating extensive resistance to various β-lactamase-encoding genes and peaking during the summer season (Table 6). STEC displayed similar resistance patterns and was often detected in conjunction with ETEC. Additionally, aEPEC showed resistance to multiple β-lactamase genes and maintained a consistent association with ETEC.

**Table 6:** The pattern of pathotypes and antibiotic resistance genes among *E. coli* isolates.

Different environmental fish groups	No. of isolates	*E. coli* pathotypes and antibiotic resistance genes
Group 1 (Winter)	3	ETEC, *bla*_*TEM*_
	1	STEC, *bla*_SHV_
	1	ETEC*, bla*_CTX-M_
	1	ETEC*, bla*_CTX-M_, *bla*_VIM_
	1	ETEC, aEPEC, *bla*_*TEM,*_* bla*_OXA-48_
	1	ETEC, *bla*_*TEM,*_* bla*_SHV,_ *bla*_OXA-48,_ *bla*_KPC_
Group 2 (Spring)	1	ETEC, *bla*_*TEM*_
	1	ETEC, *bla*_CTX-M_
	2	ETEC, STEC*, bla*_*TEM*_
	1	ETEC, *bla*_VIM_
	1	ETEC, STEC*, bla*_KPC_
	1	ETEC,* bla*_TEM,_ *bla*_KPC_
	1	ETEC, *bla*_*TEM,*_* bla*_OXA-48,_ *bla*_NDM_
	1	ETEC, *bla*_VIM*,*_* bla*_OXA-48,_ *bla*_NDM_
	1	ETEC, *bla*_CTX-M_, *bla*_SHV,_* bla*_VIM*,*_ *bla*_NDM_
Group 3 (Early Summer)	1	ETEC, *bla*_CTX-M_
	1	ETEC, STEC*, bla*_OXA-48_
	1	ETEC, *bla*_OXA-48,_ *bla*_VIM_
	1	ETEC, STEC*, bla*_CTX-M,_ *bla*_NDM_
	1	ETEC, STEC*, bla*_CTX-M ,_ *bla*_VIM_
	1	ETEC,* bla*_TEM,_ *bla*_OXA-48,_ *bla*_NDM_
	1	ETEC,* bla*_TEM,_ *bla*_SHV,_ *bla*_NDM_
	1	ETEC, STEC*, bla*_*TEM,*_* bla*_SHV,_* bla*_VIM_
	1	ETEC, *bla*_*TEM,*_* bla*_SHV,_* bla*_VIM,_ *bla*_OXA-48_
	1	ETEC, *bla*_*TEM,*_* bla*_NDM,_* bla*_VIM,_ *bla*_OXA-48_
	1	ETEC, STEC*, bla*_*TEM,*_* bla*_SHV,_* bla*_OXA-48,_ *bla*_NDM_
	1	ETEC, STEC*, bla*_*TEM,*_* bla*_SHV,_* bla*_OXA-48,_ *bla*_VIM,_ *bla*_KPC_
	1	ETEC, *bla*_*TEM,*_* bla*_SHV,_* bla*_OXA-48,_ *bla*_NDM,_ *bla*_KPC_
	1	ETEC, aEPEC*, bla*_*TEM,*_* bla*_SHV,_* bla*_OXA-48,_ *bla*_VIM,_ *bla*_NDM_
Group 4 (Summer)	1	tEPEC, *bla*_TEM_
	3	ETEC, STEC, *bla*_*TEM*_
	1	ETEC, aEPEC, *bla*_*TEM,*_* bla*_SHV_
	2	ETEC, STEC*, bla*_*TEM*_
	1	ETEC, *bla*_*TEM,*_* bla*_SHV,_ *bla*_VIM_
	1	ETEC, STEC*, bla*_*TEM,*_* bla*_SHV_
	1	ETEC, STEC*, bla*_*TEM,*_* bla*_SHV,_ *bla*_CTX-M_
	1	ETEC, *bla*_*TEM,*_* bla*_SHV,_ *bla*_VIM,_ *bla*_OXA-48_
	1	ETEC, STEC*, bla*_*TEM,*_* bla*_SHV,_ *bla*_OXA-48_
	1	ETEC, aEPEC*, bla*_*TEM,*_* bla*_SHV,_* bla*_NDM_
	1	ETEC, aEPEC*, bla*_*TEM,*_* bla*_SHV,_* bla*_OXA-48,_ *bla*_KPC_
	2	ETEC, aEPEC, *bla*_*TEM,*_* bla*_SHV,_ *bla*_CTX-M,_ *bla*_NDM,_ *bla*_OXA-48_
Group 5 (Autumn)	1	ETEC, STEC, *bla*_*TEM,*_* bla*_KPC_
	1	ETEC, STEC, *bla*_*TEM,*_* bla*_VIM_
	1	ETEC, aEPEC, *bla*_*TEM,*_* bla*_SHV_
	2	ETEC, STEC*, bla*_*TEM,*_* bla*_SHV_
	1	ETEC, *bla*_*TEM,*_* bla*_NDM,_ *bla*_CTX-M_
	1	ETEC, STEC*, bla*_*TEM,*_* bla*_VIM,_ *bla*_CTX-M_
	1	ETEC, STEC*, bla*_SHV,_ *bla*_VIM,_ *bla*_CTX-M_
	1	ETEC, STEC*, bla*_*TEM,*_* bla*_SHV,_ *bla*_NDM_
	1	ETEC, *bla*_*TEM,*_* bla*_SHV,_ *bla*_NDM,_ *bla*_VIM_
	2	ETEC, STEC*, bla*_*TEM,*_* bla*_SHV,_ *bla*_KPC,_ *bla*_OXA-48_
	1	ETEC, *bla*_*TEM,*_* bla*_SHV,_ *bla*_CTX-M,_ *bla*_NDM,_ *bla*_VIM,_ *bla*_OXA-48_
	1	ETEC, STEC*,* aEPEC, *bla*_*TEM,*_* bla*_NDM,_ *bla*_VIM,_ *bla*_OXA-48_
	1	ETEC, STEC*,* aEPEC, *bla*_SHV,_ *bla*_CTX-M,_ *bla*_NDM,_ *bla*_OXA-48_

Interestingly, one isolate from Group 4 (summer), characterized by the highest water temperature, was identified as tEPEC and linked to a β-lactamase-encoding gene. In Group 5 (autumn), under conditions optimal for *E. coli* growth (37 °C and neutral pH), some isolates exhibited a unique combination of three pathotypes, accompanied by one or more carbapenemase- or β-lactamase-producing genes.

## Discussion

This study highlights the profound impact of climate change on the prevalence, virulence, and antibiotic resistance of *E. coli* in aquatic ecosystems, particularly in aquaculture settings. The observed high prevalence of *E. coli* (92.68%) in farmed fish samples underscores the growing health risks associated with aquaculture, consistent with findings by Akter et al.^37^ in Bangladesh. Rising global temperatures and altered rainfall patterns disrupt ecological stability, creating conditions that favor the survival, proliferation, and virulence of *E. coli*^38^. The positive correlation between water temperature and *E. coli* occurrence emphasizes the critical role of temperature in shaping bacterial dynamics in fishpond environments. These findings align with Iqbal et al.^39^, who reported increased *E. coli* concentrations in warmer environments, highlighting the global significance of temperature-driven bacterial proliferation. Elevated temperatures have been linked to weakened immune responses in fish, creating favorable conditions for bacterial adaptation and spread, as previously documented by Lawlor^40^ and others^41^. Furthermore, warmer conditions not only accelerate bacterial growth but also enhance the pathogenic potential of *E. coli*, which thrives near its optimal growth temperature of 37 °C^42,43^.

The study also reveals a negative association between water pH and *E. coli* occurrence, suggesting that pH fluctuations may influence bacterial survival and virulence. These variations could be driven by factors such as biological activity, feeding practices, and daily environmental changes within controlled culture systems. Although this relationship was less pronounced compared to temperature, it aligns with the findings of Moussa et al.^44^, who reported significant impacts of pH changes on bacterial populations. The adaptability of *E. coli* to varying environmental conditions, including pH stress, underscores its resilience and ability to persist in diverse aquatic habitats^45^.

The pathogenic potential of *E. coli* is largely attributed to its virulence genes, which play critical roles in causing intestinal disorders^46^. This study identifies the dominance of *st* genes in enterotoxigenic *E. coli* (ETEC) strains, with the highest prevalence of the *st*/*lt* gene combination observed during early summer. Despite the heat-labile nature of the LT toxin, these findings suggest a complex interplay between environmental factors and strain pathogenicity^47^. Rising temperatures appear to regulate virulence through mechanisms involving heat-shock proteins and stress-responsive pathways, enhancing gene expression^48^. Similarly, the prevalence of Shiga toxin-producing *E. coli* (STEC) peaked during warmer months, with the *stx2* gene being more common than *stx1*, indicating higher virulence potential^49^. The temperature-regulated virulence of enteropathogenic *E. coli* (EPEC), particularly atypical EPEC (aEPEC), further emphasizes the role of environmental factors in modulating pathogenicity. This study reveals that aEPEC strains are more prevalent than typical EPEC (tEPEC), with their occurrence peaking during summer at 39 °C. The activation of virulence genes in aEPEC, attributed to the locus of enterocyte effacement (LEE), involves temperature-sensitive regulatory cascades, such as those mediated by the Ler protein^50^.

The emergence of hybrid *E. coli* pathotypes, which harbor virulence markers from multiple pathotypes, presents significant challenges for disease management due to their enhanced pathogenic potential and resistance to treatment. The genetic flexibility of *E. coli* facilitates horizontal transfer of virulence factors, leading to hybrid strains that defy traditional classification^51,52^. Climate change exacerbates this phenomenon by fostering bacterial proliferation, transmission, and horizontal gene transfer under elevated temperatures, as documented by Zhao et al.^53^.

Aquatic environments are recognized as critical reservoirs for antibiotic-resistant bacteria (ARB), with improper antibiotic use, contamination, and aquaculture practices driving AMR development^21,54^. Rising temperatures amplify these issues by promoting bacterial adaptation and stress responses^55^. Bacteria develop antibacterial resistance through various mechanisms, including enzyme production (e.g., β-lactamases and carbapenemases) that degrade antibiotics, efflux pumps that expel drugs, and target site modifications that prevent antibiotic binding. Changes in membrane permeability reduce drug entry, while biofilm formation protects bacteria from antimicrobials and immune responses. Additionally, resistance genes spread via horizontal gene transfer through plasmids, transposons, or bacteriophages, making treatment more challenging^56^.

This study highlights the detection of extended-spectrum β-lactamase (ESBL)-encoding genes in *E. coli* isolates from aquaculture, particularly in warmer waters. The predominance of the *bla*_TEM_ gene over other ESBL genes, such as *bla*_SHV_ and *bla*_CTX-M_, aligns with previous reports^57^. Interestingly, *bla*_SHV_ exhibited a temperature-dependent prevalence, while *bla*_CTX-M_ showed variable distribution across different environmental conditions, with the highest rate observed in autumn. Additionally, isolates from summer and autumn frequently harbored multiple ESBL genes, consistent with findings by Silago et al.^58^, who documented the co-occurrence of *bla*_CTX-M_, *bla*_TEM_, and *bla*_SHV_ in bacterial isolates.

Carbapenemase-encoding genes, including *bla*_OXA-48_, *bla*_NDM_, and *bla*_VIM_, were prominent during early summer, summer, and autumn. Among them, *bla*_OXA-48_ was the most frequently detected, aligning with previous reports from Iraq^59^. The prevalence of *bla*_NDM_ and *bla*_VIM_ significantly increased in early summer at 35 °C and in autumn at 37 °C, suggesting that elevated temperatures create favorable conditions for bacterial survival and proliferation^60^. Notably, the highest frequency of *bla*_KPC_ was recorded at 37 °C in autumn, further emphasizing the role of increased temperatures in promoting the clonal spread of carbapenemase genes.

The coexistence of β-lactamase and carbapenemase genes in *E. coli* isolates represents a significant challenge for treatment, as this combination confers resistance to a wide range of antibiotics, potentially leading to multidrug-resistant infections^61^. The diversity of resistance gene combinations observed, particularly during warmer months, underscores the influence of environmental factors such as temperature and pH on the development of antibiotic resistance in aquaculture.

These findings underline the urgency of addressing climate change to mitigate the rapid dissemination of antibiotic-resistant genes in aquatic ecosystems. Environmental factors contribute to the spread of antimicrobial-resistant *E. coli*, increasing health risks associated with consuming contaminated fish^62^.

The findings also reveal a strong association between *E. coli* pathotypes, such as ETEC, aEPEC, and STEC, and resistance genes. These pathotypes frequently co-occurred, particularly during warmer months. Such combinations not only complicate the treatment of infections but also pose a risk of zoonotic transmission through the food chain or direct contact with contaminated water^63^.

*Escherichia coli* serves as a key indicator of water quality, highlighting potential sewage contamination and fecal pollution^64^. The presence of pathogenic and antibiotic-resistant strains in water bodies poses significant risks to both aquatic life and public health. Regular monitoring and strict water quality management are essential to mitigate these risks and prevent the spread of resistant infections^65,66^. Additionally, further studies on water samples are recommended to better understand their impact on fish health and public safety.

## Conclusion

This study provides critical evidence that environmental factors, particularly rising temperatures and pH fluctuations, significantly influence the prevalence, virulence, and antibiotic resistance of *E. coli* in aquaculture. The identification of diverse diarrheagenic *E. coli* pathotypes, hybrid strains, and their associated virulence genes underscores the growing public health threat posed by these bacteria, especially in warmer conditions. Additionally, the widespread presence of β-lactamase- and carbapenemase-encoding genes highlights the alarming escalation of antibiotic resistance, with the coexistence of these resistance determinants complicating treatment and fostering multidrug-resistant strains.

These findings emphasize the urgent need for continuous surveillance of aquaculture environments, stricter regulations on antibiotic use, and proactive measures to mitigate climate change’s impact on aquatic ecosystems. Addressing these challenges is crucial for safeguarding food safety, public health, and the sustainability of aquaculture.

## Data Availability

All the data generated or analyzed in this study are included in this published article.
